# iTRAQ-based quantitative proteomic analysis provides insight into the drought-stress response in maize seedlings

**DOI:** 10.1038/s41598-022-13110-7

**Published:** 2022-06-09

**Authors:** Wen Ren, Zi Shi, Miaoyi Zhou, Bingbing Zhao, Hanshuai Li, Jiarong Wang, Ya Liu, Jiuran Zhao

**Affiliations:** Maize Research Institute, Beijing Academy of Agriculture and Forestry Sciences, Beijing Key Laboratory of Maize DNA Fingerprinting and Molecular Breeding, Shuguang Garden Middle Road No. 9, Beijing, 100097 China

**Keywords:** Agricultural genetics, Gene expression, Gene regulatory networks, Proteome informatics, Plant stress responses

## Abstract

Drought is a major abiotic stress that harms plant cell physiology and limits the growth and productivity of crops. Maize (*Zea mays* L.), one of the most drought-susceptible crops, is a major food source for humans and an important resource for industrial bioenergy production; therefore, understanding the mechanisms of the drought response is essential for maize improvement. Using isotopic tagging relative quantitation (iTRAQ)-based protein labeling technology, we detected the proteomic changes in maize leaves under drought stress. Among the 3063 proteins that were identified, the abundance of 214 and 148 proteins increased and decreased, respectively, after three days of drought treatment. These differentially abundant proteins (DAPs) were mainly involved in cell redox homeostasis, cell wall organization, photosynthesis, abscisic acid biosynthesis, and stress-response processes. Furthermore, some of the DAP abundances still differed from the control six days after the drought treatment, most of which were molecular chaperones, heat shock proteins, metabolism-related enzymes, hydrolases, and transmembrane signal receptors. The expression level of some DAPs returned to normal when the water supply was restored, but for others it did not. A significant correlation between the protein and transcript levels was observed following an RT-qPCR analysis. Finally, our research provides insights into the overall mechanism of drought-stress tolerance, and important information for breeding of drought-tolerant maize.

## Introduction

The growth and yield of crops are strongly affected by the environment. Drought, a complex environmental issue caused by reduced rainfall, decreased soil moisture, and increased temperatures, occurs in almost all climatic regions, and is one of the major stress factors responsible for yield losses^1,2^. As global warming intensifies, more than 20% of land will be affected by drought, especially in major maize (*Zea mays* L.)-producing areas in East Asia, South America, and Western Europe^3^. It is necessary to increase crop yields despite the changing climate to ensure the provision of food to the growing global population. Through breeding efforts and technological improvements, many drought-tolerant crop varieties have been developed; however, global climate change and uncertainty in precipitation patterns may lead to long-term drought stress, which will likely still reduce yields^2^.

Under drought stress, the growth of plants is greatly inhibited and various physiological and morphological responses are observed^4^. Drought reduces plant leaf area, root density, and biomass accumulation. Plants have evolved some strategies to cope with drought stress however; for example, drought-stressed plants can maintain the water status and physiological activity of their cells using an abnormal accumulation of metabolites (such as sugars, betaines, polyamines, and amino acids, particularly proline and glycine)^5,6^. In response to drought stress, plant cells biosynthesize and accumulate a large number of drought tolerance-related proteins (molecular chaperones, late embryogenesis abundant (LEA), aquaporins, and antioxidant defense enzymes), which constitutes an important molecular response to drought stress^7^. In order to ensure food security, it is necessary to identify and understand the molecular mechanisms of drought stress on plant development and physiology, enabling the further improvement of crop drought tolerance in the future.

Maize is the third most cultivated cereal crop in the world, providing food for humans and animals in addition to its use in various industrial applications including bioenergy production. Maize is highly productive under appropriate growth conditions; however, in many parts of the world, it is grown in semi-arid environments characterized by a combination of water shortage and high temperatures. Although maize originated in the tropics, it is still sensitive to drought and high temperatures, especially after entering the eight-leaf stage^8^; for example, at a soil moisture content of 40%, maize yields decrease by 39%^9^. At present, China cultivates about 42 million hectares of maize (https://data.stats.gov.cn/search. htm); however, about 60% of this area is semi-arid, with a maize yield loss of 20–30% per year^10^.

In a previous study of the drought-stress response of maize plants at the three-leaf stage, we identified several genes putatively involved in this process using RNA-seq, as well as providing useful resources for the investigation of the specific functions of these drought-responsive genes in maize^11^. Although RNA-seq is often used to study gene expression differences under different conditions, the mRNA expression level does not always directly correspond to the abundance of the corresponding protein. The levels of proteins not only depends on the rate of transcription, but also on mRNA nuclear export, mRNA stability, translation regulation, and protein degradation^12^. Isotopic tagging relative quantitation (iTRAQ) has been proposed to represent a more accurate and reliable method for the quantitation of proteins^13^. Many researchers have used the iTRAQ technique to study the tolerance of crops under various stress conditions, such as osmotic stress, aluminum stress, and high temperatures^14–16^.

In order to reduce the effect of drought stress on maize growth, we carried out research on drought stimuli in the early growth stage. We want to find some functional proteins involved in maize drought stress by detecting the change of proteins under drought conditions. It’s will be helpful to provide candidates for improving the growth and yield under drought environment. In this study, we analyzed the changes in the protein levels of maize subjected to drought and rewatering using an iTRAQ-based quantitative proteomic analysis. Furthermore, we utilized bioinformatic analyses to confirm the functions of the differentially abundant proteins (DAPs) in the maize drought response. Many of the DAPs were found to be related to cell redox homeostasis, cell wall organization, photosynthesis, abscisic acid biosynthesis, and stress-response processes. Our results provide a basis for the further elucidation of the molecular mechanisms underlying the response to drought stress in maize.

## Results

### Primary data analysis and protein identification

**Figure 1:**
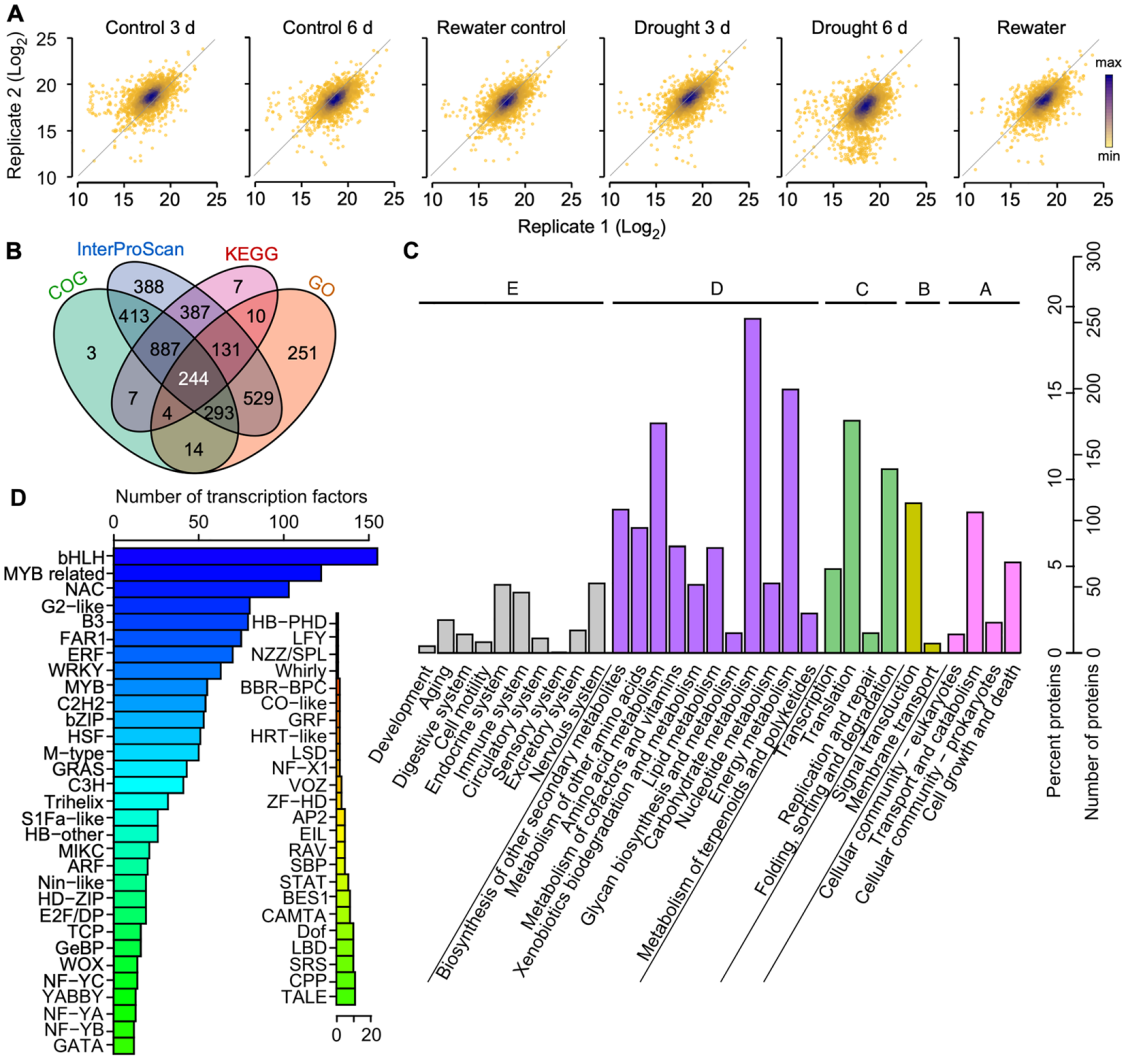
Basic iTRAQ output details. (**A**) Scatter plots showing the correlation between two replicates for different conditions. (**B**) Venn diagrams of the identified peptides using different annotation techniques. (**C**) The KEGG annotation for the identified peptides. (**D**) Frequency of each transcription factor class in the identified peptides.

First, we tested the repeatability of two samples at the same timepoint, demonstrating that our results have good repeatability and confirming the credibility of the data (Fig. 1A). By integrating all the data, we identified a total of 3063 proteins from all the 12 samples and annotated them using different databases. The Kyoto Encyclopedia of Gene and Genomes (KEGG) is a database resource of understanding of high-level functions and utilities of the biological system. The Clusters of Orthologous Groups of proteins (COG) database contains phylogenetic classification of proteins encoded in complete genomes. The Gene Ontology (GO) database is the largest source of information on the functions of genes. The InterPro provides functional analysis of proteins by classifying them into families and predicting domains and sites (Fig. 1B). We further analyzed the function of these proteins using a KEGG annotation, revealing that these identified proteins are involved in various metabolic processes. The most highly enriched pathways were carbohydrate metabolism, amino acid metabolism, energy metabolism, and mRNA translation (Fig. 1C). These data reveal the metabolic changes in the plant cells in response to the water-limited growth conditions. In consideration of the important role of transcription factors in the regulation of various life activities, we classified the detected transcription factors, revealing that the most abundant categories included bHLHs, MYBs, NACs, and G2-like proteins (Fig. 1D).

### Analysis of drought-responsive DAPs after a 3-d drought

**Figure 2:**
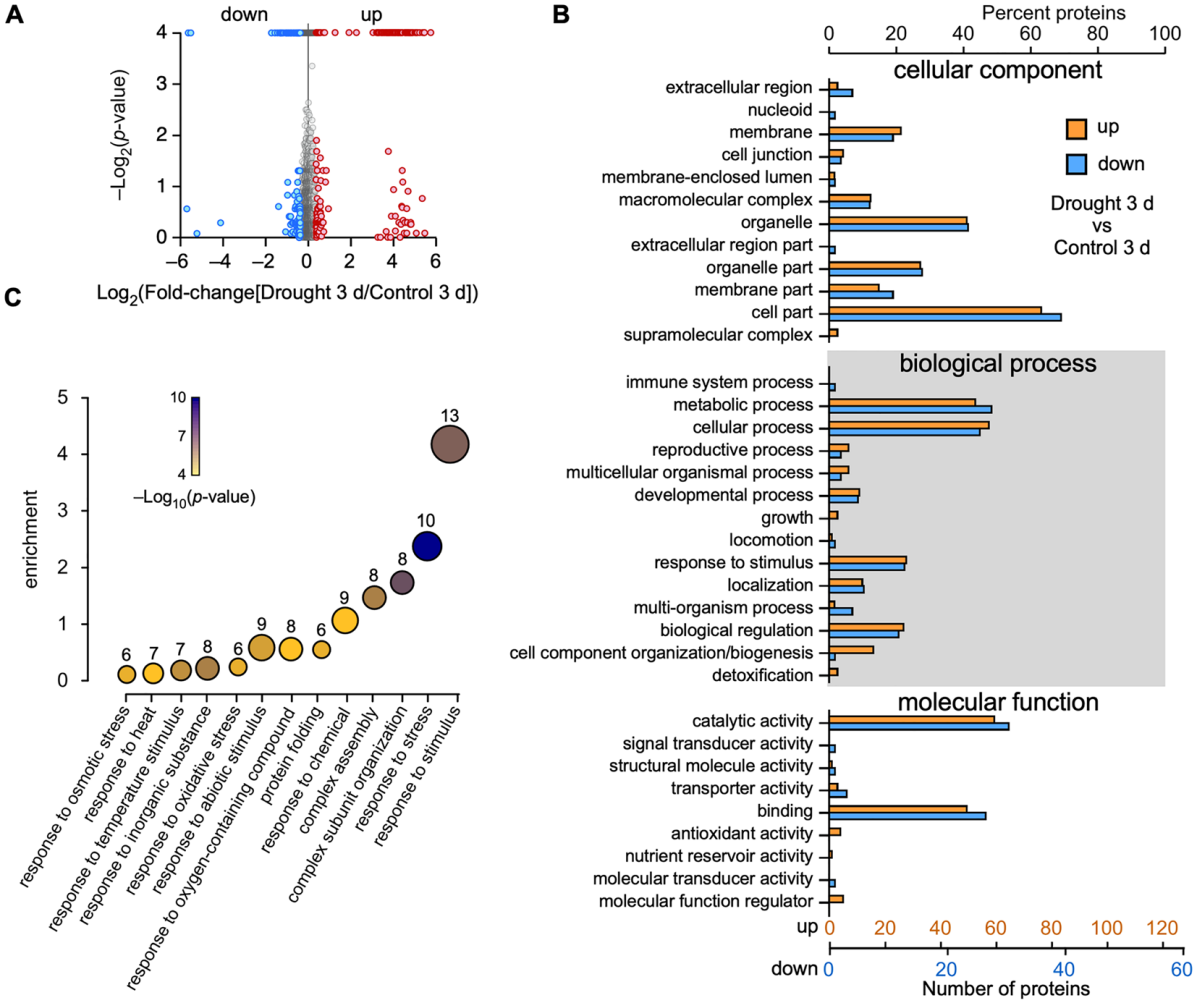
DAPs involved in drought-tolerance pathways. (**A**) Volcano map showing the DAPs identified following the 3-d drought treatment. (**B**) GO annotation for all DAPs. (**C**) GO enrichment analysis of the upregulated proteins after the 3-d drought.

In order to explore the factors with important effects on the drought response, we first analyzed the samples after 3 d of drought treatment. We identified a total of 362 DAPs between the control and experimental plants subjected to a 3-d drought, including 214 upregulated and 148 downregulated proteins (Fig. 2A, Tables S1 and S2). Gene ontology (GO) annotations were used to assign and analyze the pathway enrichment of the DAPs, which were classified into 35 GO terms (Fig. 2B). The most common terms in cellular components are organelles and cell parts. The most enriched terms in biological process category are metabolic processes, cellular processes, responses to stimuli, and biological regulation, while the most enriched terms in molecular function category are catalytic activity and binding. (Fig. 2B). Moreover, a further analysis of the enriched GO terms (p-value < 0.05) revealed that many of the up-regulated proteins are involved in the responses to stimuli and stress (Fig. 2C). Meanwhile, there are some enriched GO terms related to responses to stimuli and pigment metabolism for down-regulated proteins, which may suggest that these proteins are involved in photosynthesis (Figure S1A).

Flavonoids are a large class of low-weight phenols, which play vital roles in various biochemical and physiological processes of plants, such as UV protection, defense against pathogens, and drought stress^17,18^. We showed that some of the DAPs (eg. shikimate O-hydroxycinnamoyl transferase and flavonoid 3'-monooxygenase) are involved in the flavonoid biosynthesis and oxidative phosphorylation pathways (Figure S1B). These data suggested that drought stimuli can affect flavonoid biosynthesis and the oxidative phosphorylation pathway.

### Comparing the DAPs between plants after 3-d and 6-d droughts

**Figure 3:**
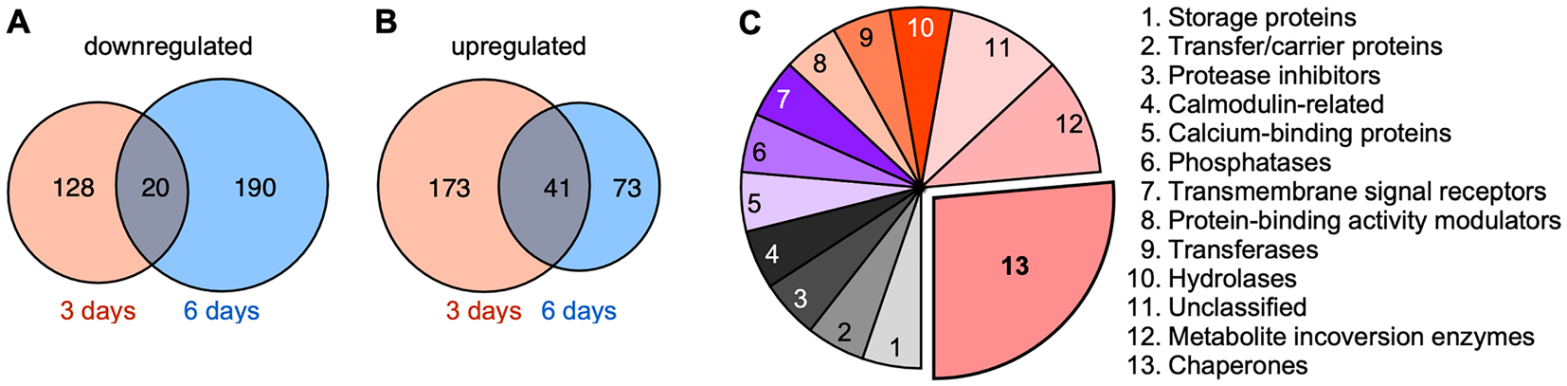
Comparison of the DAPs after a 3-d and 6-d drought. (**A**) Venn diagram illustrating the overlap between the downregulated proteins in the plants subjected to 3-d and 6-d drought treatments. (**B**) Venn diagram illustrating the overlap between the upregulated proteins in the plants subjected to 3-d and 6-d drought treatments. (**C**) Categorization of the 41 proteins up-regulated in both the 3-d and 6-d drought treatments.

We further compared the DAPs in the plants subjected to 3-d and 6-d droughts versus the corresponding control, respectively. A total of 20 and 41 DAPs were commonly downregulated and upregulated, respectively, in the plants subjected to 3-d and 6-d droughts (Fig. 3A,B and Table S3). These overlapping DAPs may therefore be important for the drought response, so we further analyzed the function of the overlapping proteins. Some of these up-regulated DAPs were annotated as metabolite interconversion enzymes, chaperones, and transferases (Fig. 3C). Furthermore, some of the down-regulated DAPs are annotated as transporter, scaffold protein and DNA metabolism proteins (Figure S1C).

Drought-responsive proteins such as dehydrins and heat shock proteins (HSPs) are produced to protect the intracellular metabolic machinery. The dehydrin COR410 localizes to the plasma membrane and has been reported to accumulate under water stress in wheat (*Triticum aestivum*)^19^. Here, we found that COR410 was upregulated in plants after 3 d of drought, suggesting that it also takes part in the drought response in maize. The HSPs take part in various stress responses, not just heat stress. Six of the 41 members of the HSP20 family were found to be upregulated in the droughted plants in the present study, indicating that the HSP20 family may play a crucial role in the drought-stress response in maize. The abundance of proteins related to cell wall biogenesis and degradation, such as glucan endo-1,3-beta-glucosidase 7, also significantly increased in the droughted samples. Some of the other DAPs were also involved in this metabolic pathway, such as glutamate decarboxylase isoform X2 and GDP-fucose protein O-fucosyltransferase. Some DAPs have a currently unknown function, and may represent new regulatory targets for further drought research.

### Analysis of DAPs after rewatering

**Figure 4:**
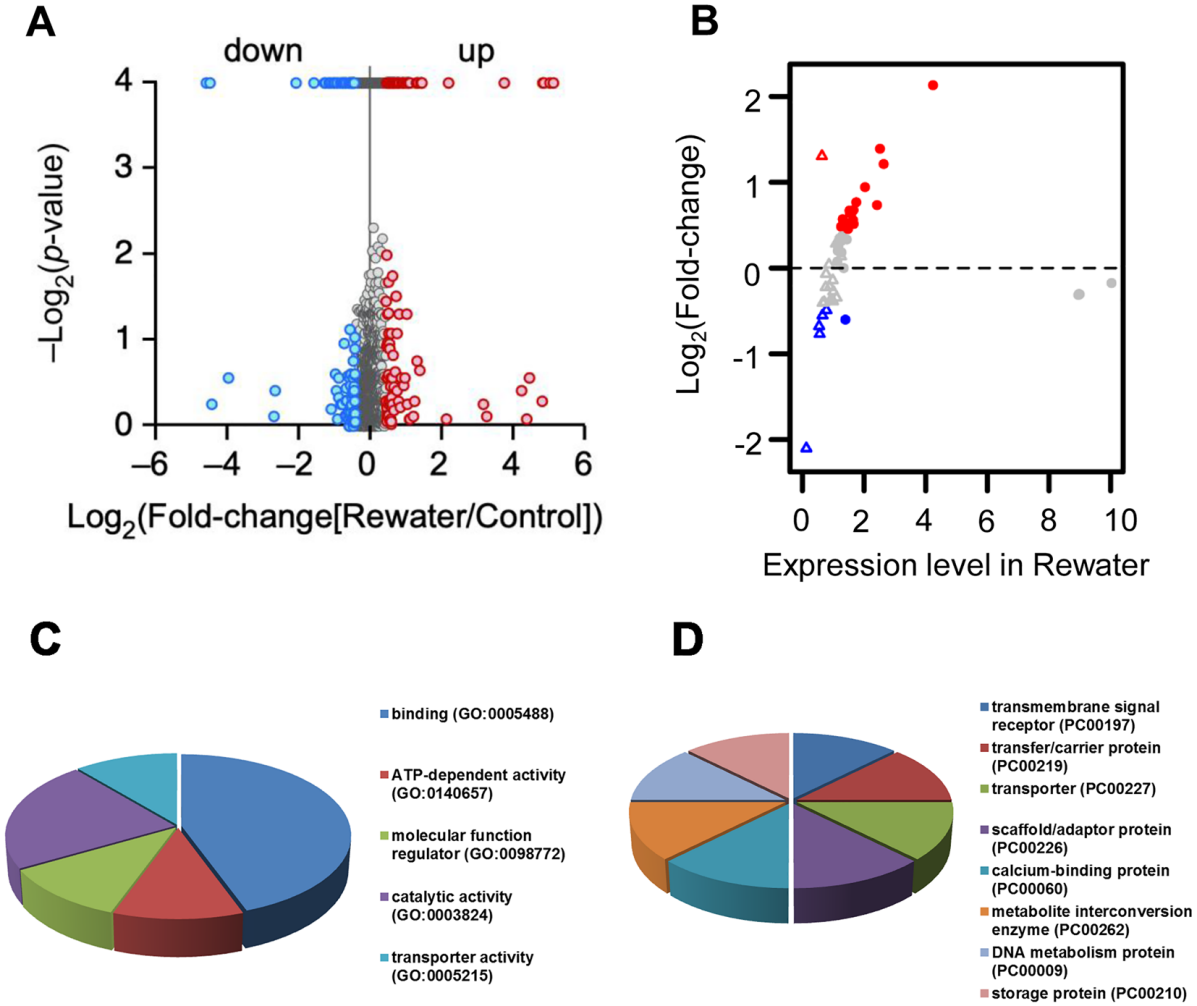
The change in drought-response proteins after rewatering. (**A**) Volcano map showing the DAPs identified following rewater treatment. (**B**) Scatter plots showing the change in abundance of the DAPs from the drought treatments following the rewatering. Red indicates an upregulation following rewatering, while blue represents a downregulation. Circle represents a protein upregulated in the droughted plants, while a triangle represents a downregulated protein. (**C**) GO annotation for the recovery proteins. (**D**) Function analysis for the recovery proteins.

To further explore whether the DAPs returned to normal levels when the water supply returned, we analyzed the protein levels of samples rehydrated for 1 d following a 6-d drought in comparison with the control samples. The maizes were still alive after 6 d of drought treatment, but some leaves appeared withered. We found a total of 231 DAPs, of which 127 were upregulated and 104 were downregulated (Fig. 4A). Some of the DAPs under the drought treatment returned to the same abundance as the control when the water supply was restored (Fig. 4B, Table S5), suggesting that the expression of these proteins is most sensitive to drought stimulation. GO analyzed indicated that some of these DAPs belong to binding proteins and have the ATP-dependent activity, suggested that these proteins may take part in signal transduction (Fig. 4C and D).By contrast, some of the DAPs did not recover to normal levels after the water supply was resumed.

### Cluster analysis of the change trend of the DAPs during drought and rewatering

**Figure 5:**
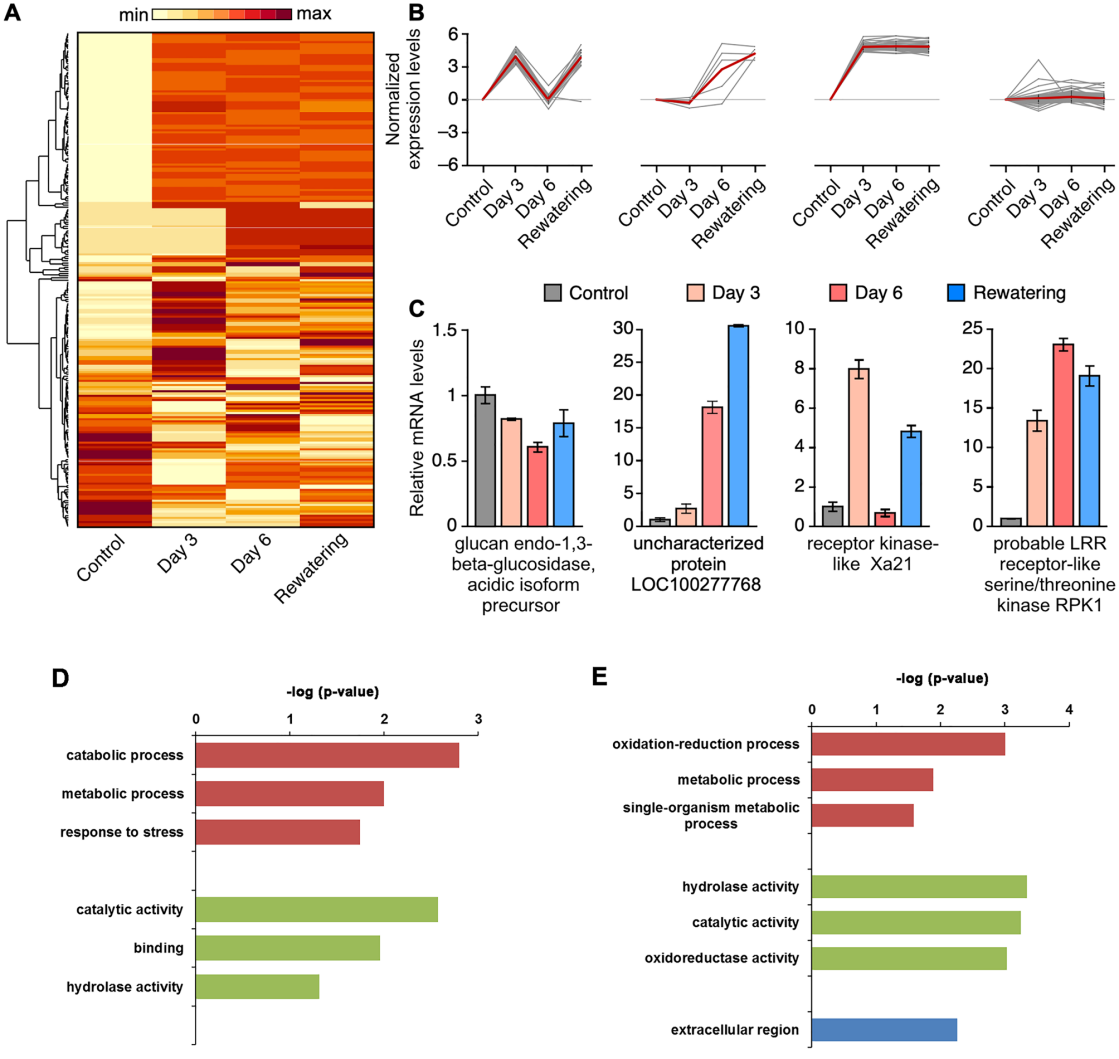
Cluster analysis of the DAPs during the drought treatments. (**A**) Heat map illustrating the expression profiles of the DAPs at three timepoints (3-d drought, 6-d drought, and rewatering). (**B**) Four enriched clusters of the gene expression profiles determined using the K-means algorithm. (**C**) Randomly selected DAPs were analyzed using RT-qPCR. (**D**) GO enrichment result of Cluster 3 proteins. The red color represent BP (biological process), the green color represent MF (molecular function) and the blue color represent CC (cellular component). (**E**) GO enrichment result of Cluster 4 proteins. The red color represent BP (biological process), the green color represent MF (molecular function) and the blue color represent CC (cellular component).

We performed a cluster analysis on the change trend of the DAPs at different samples and found that some proteins were upregulated under the stimulation of drought (Fig. 5A). Through time series clustering, we found that the expression levels of the DAPs showed four major trends during the drought treatment and rewatering: there was no obvious rule in the Cluster 1 DAPs; the Cluster 2 DAPs gradually increased throughout the drought and rewatering; the Cluster 4 DAPs peaked after the 3-d drought treatment then remained high; while the Cluster 3 DAPs present a wave-like change, increasing after 3 d of drought, decreasing after 6 d of drought, then finally increasing again after the rewatering (Fig. 5B).

We detected the transcriptional changes in the genes encoding DAPs using RT-qPCR. For the randomly selected 16 DAPs, the mRNA changes were consistent with the protein change trend for 8 DAPs, indicating that maize regulated the expression changes of these proteins at the transcriptional level under drought stimuli (Fig. 5C). By contrast, there are still mRNA changes were inconsistent with the protein changes for another 8 DAPs (data not shown), indicating that the regulation of the abundances of these proteins may occur at the protein translation or stability levels.

To explore the function of these different cluster proteins, we using GO analyze to annotate each cluster. There were not enrichment terms for Cluster 1 and Cluster 2. Some of the Cluster 3 proteins are take part in catabolic process and metabolic process, and the proteins in Cluster 4 are involved in oxidation–reduction process and metabolic process (Fig. 5D and E).

## Conclusion

In this study, we used iTRAQ-based proteomics to investigate the response of maize to drought stress on a proteome scale. In comparison with the control conditions, 214 proteins were upregulated and 148 proteins were downregulated after a 3-d drought. A bioinformatic analysis showed that these DAPs were mainly involved in cell redox homeostasis, cell wall organization, photosynthesis, abscisic acid biosynthesis, and stress-response processes. Among the DAPs, we found that a large portion of these proteins were encoded by the chloroplasts or mitochondria, suggesting that drought stress strongly affects the function of these organelles in maize.

HSPs act as chaperones for a variety of proteins in response to various stresses, playing key roles in protecting plants. Our proteomics data show that several HSP20 family members were more abundant under drought stress, indicating that this family may be important for the maize drought response. Not all HSPs increased in abundance however; some HSPs even decreased under drought stress. HSP70 was previously shown to decrease in abundance in drought-treated rapeseed (*Brassica napus* subsp. *napus*)^20^, while the levels of HSP17 and three HSP70 proteins decreased in wheat^21^. The functions of the HSPs in plant drought tolerance are therefore worthy of further study.

Throughout our experiments, we found that maize also responded to stress by reducing the abundance of many proteins. In higher plants, isoprenoids constitute a large class of natural products playing important roles in plants. isoprenoid compounds are biosynthesized from the C5 precursor isopentenyl diphosphate and its isomer dimethylallyl diphosphate^22,23^. We found that the abundance of enzymes involved in isoprenoid biosynthesis, isopentenyl-diphosphate delta-isomerase I and 1-deoxy-d-xylulose 5-phosphate reductoisomerase, decreased under drought stress. Previous studies showed that the levels of mRNAs encoding 1-deoxy-d-xylulose 5-phosphate synthase and isoprene diphosphate isomerase decreased under drought^24^, which is consistent with our finding.

Plants reduce their photosynthesis and respiration rates to cope with drought stress. We found that the expression of some proteins involved in these physiological processes decreased in droughted plants, such as NADPH-dependent aldehyde reductase-like protein, NADP-dependent oxidoreductase P2, NAD(P)H-quinone oxidoreductase subunit 4L, and the tetratricopeptide repeat domain-containing protein PYG7. We also detected changes in some epigenetic factors, such as the Probable ATP-dependent DNA helicase CHR12 chromatin remodeler. These results suggested that epigenetic regulation may also be involved in the stress response of maize to water scarcity.

It is worth noting that the expression level of some proteins returned to normal when the water supply was restored, but for others it did not after rehydration for 1 day. The impact of water shortage on plants may therefore have long-term effects, and rewatering cannot completely restore them to a normal physiological level.

In conclusion, our results provide us with a better understanding of the physiological and molecular responses to drought stress in maize, and may provide an insight into the mechanisms underlying drought-stress tolerance. We did not explore changes in post-translational protein modifications, such as phosphorylation, methylation, and acetylation, which are known to play important roles in regulating cell signaling^25,26^. The future detection of protein modifications will therefore provide new insights into the drought-tolerance mechanisms of maize.

## Materials and methods

### Plant materials, growth conditions, and drought stress treatments

The seeds of the maize inbred line B73 were preserved in the Maize Research Institute of Beijing Academy of Agricultural and Forest Sciences (BAAFS) in Beijing, China. The seeds were planted and subjected to drought-stress treatment, as described previously^11^. Briefly, the seedlings were grown at 25 ± 2 °C and 60–70% humidity, with an 18-h photoperiod provided by natural sunlight. A total of 12 pots of samples were equally divided into two groups: an experimental group and a control group. The experimental group consisted of three time points: 3 d of drought, 6 d of drought, and 1 d of rehydration after 6 d of drought, with two replicate samples at each timepoint; the control group was sampled at the same timepoints. Yellow and brown leaf tips were removed before the collected samples were frozen in liquid nitrogen.

### iTRAQ labeling and fractionation

#### iTRAQ labeling and liquid chromatography (LC)–mass spectrometry (MS)/MS proteomic analysis

The samples were labeled with an iTRAQ reagent eight-plex kit (Thermo Fisher Scientific, Waltham, MA, USA), according to the manufacturer’s protocol. Two biological replications were performed for each timepoint, with a total of 12 samples prepared for quantitative proteomics experiments. After labeling, all samples were mixed and purified with a strong cation exchange chromatography column using an Agilent 1200 high-performance LC system (Agilent Technologies, Santa Clara, CA, USA) and separated using LC using an Eksigent nanoLC-Ultra 2D system (AB Sciex, Framingham, MA, USA).

#### Protein identification and quantification

The LC–MS/MS spectra were searched using the MASCOT engine (Matrix Science, London, UK; version 2.2) embedded in Proteome Discoverer 1.4 (Thermo Fisher Scientific), and run against the maize protein database (http://ftp.maizegdb.org/MaizeGDB/FTP/maize_proteome/proteome.fasta). The DAPs were identified using ProteinPilot (AB Sciex). The abundance ratios were used to assess the fold changes in the abundance of the proteins identified in the drought-treated plants compared with the control plants. The DAPs were defined as those showing at least a 1.5-fold change (adjusted *p* ≤ 0.05) relative to their corresponding controls.

### RT-qPCR verifying

Total RNA was extracted from seedling shoots using Trizol reagent (Thermo Fisher Scientific). The extracted total RNA was treated with RQ1 RNase-free DNase (Promega, Madison, WI, USA), after which first-strand cDNA was amplified using M-MLV Reverse Transcriptase (Promega). The RT-qPCR was completed using an ABI 7500 Real-Time PCR System (Thermo Fisher Scientific) and SYBR Premix (Thermo Fisher Scientific). Two independent experiments were performed, each with three technical replicates. The relative expression was calculated as previously described^27^.The results of a representative experiment are provided, with the data presented as the mean ± SD (n = 3).

### GO annotation and Mapman gene clustering

A GO enrichment analysis was performed on the differentially expressed protein sets using the agriGO online tool (http://bioinfo.cau.edu.cn/agriGO/). Significant GO terms (q ≤ 0.05) were selected. A pathway enrichment analysis of the different sets of proteins was performed using MapMan (v 3.5.1R2) (https://mapman.gabipd.org/), The up- and downregulated proteins are indicated in red and green, respectively. For the cluster classification, the DAPs were grouped into four clusters using the K-means method. For a given set of proteins, the row-wise Z-scores were determined and a heatmap was generated using the heatmap2 package in R. The volcano maps were also generated using an R package.

### Ethical approval

Experimental research in this study on plants in this study, including the collection of plant material, complies with relevant institutional, national, and international guidelines and legislation. The permission of maize seed collection was obtained.

## Supplementary Information

Supplementary Legends.

Supplementary Figure S1.

Supplementary Table S1.

Supplementary Table S2.

Supplementary Table S3.

Supplementary Table S4.

Supplementary Table S5.

## Data Availability

The data presented in this study are available on request from the corresponding authors.
