# Supplementary material for: iTRAQ-based quantitative proteomic analysis provides insight into the drought-stress response in maize seedlings
Source: Sci Rep. 2022 Jun 9;12:9520. doi: 10.1038/s41598-022-13110-7 (PMC9184573; doi:10.1038/s41598-022-13110-7)
Supplement: Supplementary file 1 — Supplementary Legends. [file 41598_2022_13110_MOESM1_ESM.docx]

Table S1. Upregulated proteins after a 3-d drought

Table S2. Downregulated proteins after a 3-d drought

Table S3. Overlapping proteins in plants subjected to a 3-d or 6-d drought

Table S4. Primers used for RT-qPCR

Table S5. Recovery proteins after re-watering.

Figure S1. The function of the DAPs.

(A) GO enrichment analysis of the down-regulated proteins after the 3-d drought.

(B) The changed DAPs of flavonoids pathway.

(C) Categorization of the 20 proteins down-regulated in both the 3-d and 6-d drought treatments.
