# Supplementary material for: iTRAQ-based quantitative proteomic analysis provides insight into the drought-stress response in maize seedlings
Source: Sci Rep. 2022 Jun 9;12:9520. doi: 10.1038/s41598-022-13110-7 (PMC9184573; doi:10.1038/s41598-022-13110-7)
Supplement: Supplementary file 2 — Supplementary Figure S1. [file 41598_2022_13110_MOESM2_ESM.pdf]

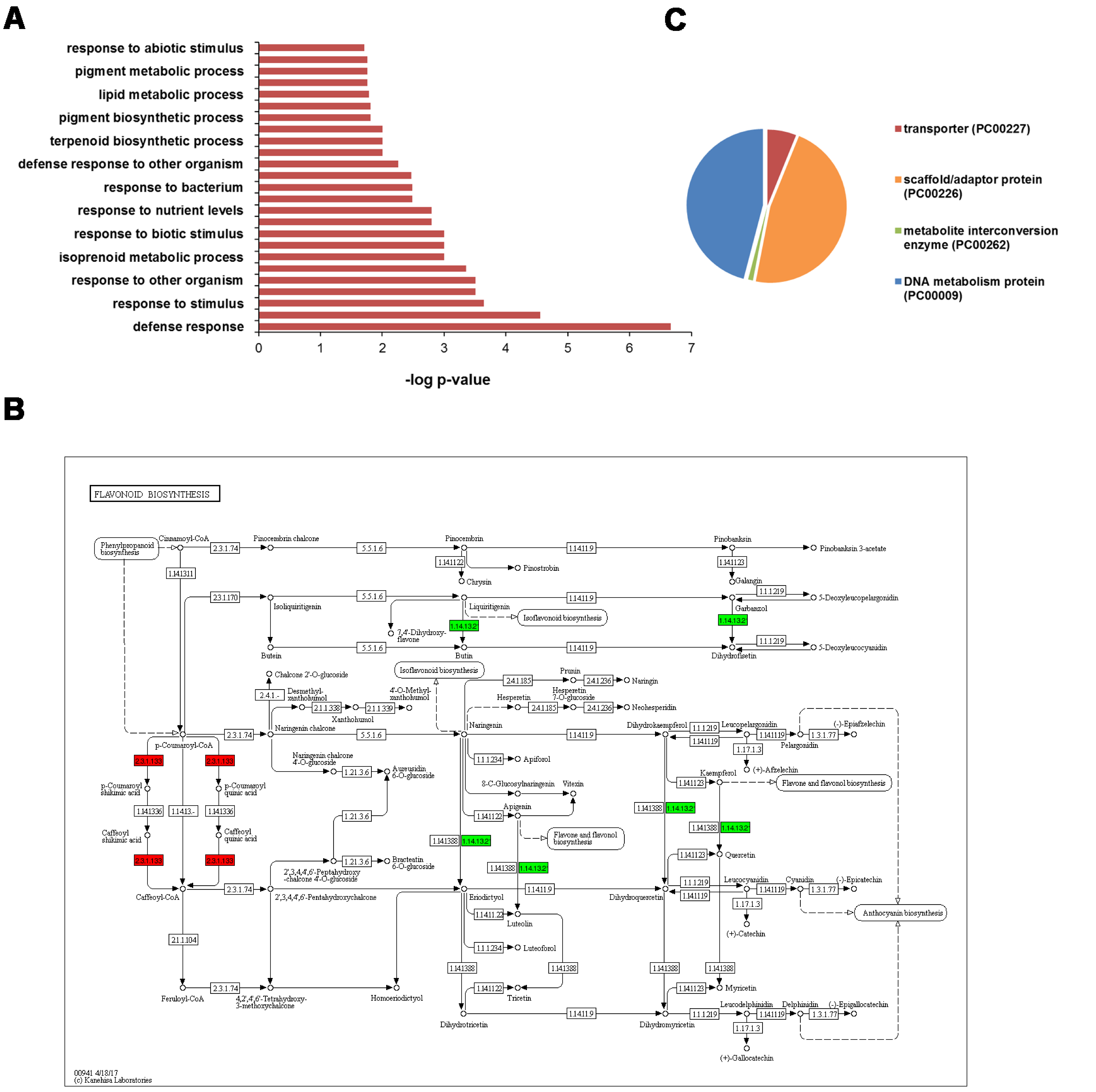

Figure S1. The function of the DAPs.(A) GO enrichment analysis of the down-regulated proteins after the 3-d drought. (B) The changed DAPs of flavonoids pathway.(C) Categorization of the 20 proteins down-regulated in both the 3-d and 6-d drought treatments.
